# Hysteresis-free perovskite solar cells made of potassium-doped organometal halide perovskite

**DOI:** 10.1038/s41598-017-12436-x

**Published:** 2017-09-22

**Authors:** Zeguo Tang, Takeru Bessho, Fumiyasu Awai, Takumi Kinoshita, Masato M. Maitani, Ryota Jono, Takurou N. Murakami, Haibin Wang, Takaya Kubo, Satoshi Uchida, Hiroshi Segawa

**Affiliations:** 1 Research Center for Advanced Science and Technology (RCAST), The University of Tokyo, 4-6-1, Komaba, Meguro-ku, Tokyo, 153-8904 Japan; 2 Graduate School of Arts and Sciences, The University of Tokyo, Komaba 3-8-1, Meguro-ku, Tokyo, 153-8902 Japan; 3 Research Center for Photovoltaics, National Institute of Advanced Industrial Science and Technology (AIST), 1-1-1 Higashi, Tsukuba, Ibaraki, 305-8565 Japan

## Abstract

Potassium-doped organometal halide perovskite solar cells (PSCs) of more than 20% power conversion efficiency (PCE) without *I-V* hysteresis were constructed. The crystal lattice of the organometal halide perovskite was expanded with increasing of the potassium ratio, where both absorption and photoluminescence spectra shifted to the longer wavelength, suggesting that the optical band gap decreased. In the case of the perovskite with the 5% K^+^, the conduction band minimum (CBM) became similar to the CBM level of the TiO_2_-Li. In this situation, the electron transfer barrier at the interface between TiO_2_-Li and the perovskite was minimised. In fact, the transient current rise at the maximum power voltages of PSCs with 5% K^+^ was faster than that without K^+^. It is concluded that stagnation-less carrier transportation could minimise the *I-V* hysteresis of PSCs.

## Introduction

Organometal halide perovskites have captured wide interest as a promising material for low-cost and high-efficiency solar cells^1–4^. The power conversion efficiency (PCE) of the organometal halide perovskite solar cells (PSCs) has reached 22% within a few years of its advent^5^, which is comparable to those of polycrystalline Si solar cells (21.9%)^6^, CdTe solar cells (22.1%)^7^, and CIGS solar cells (22.6%)^8^. Through recent studies of PSCs, the composition of organometal halide perovskites is recognised as one of the key factors in the improvement of the PCE. As for the organic cation methylammonium (MA), the mixing of formamidinium (FA) extended the absorption edge from 800 nm to 850 nm, and then the photocurrent of PSC was enhanced^9–11^. However, the pure formamidinium lead iodide (FAPbI_3_) contained the non-perovskite phase (δ-phase), which resulted in the low PCE of the perovskite solar cells^12–15^. To suppress the creation of the δ-phase, the methylammonium chloride or bromide (MACl or MABr, respectively) was incorporated into the perovskite layer^12,16,17^, and the PCE was improved to more than 20%^18^. It was also verified that the incorporation of ***caesium*** cations (Cs^+^) into the A-site of perovskite inhibited the creation of the δ-phase, which considerably promoted the stability of the perovskite phase and the reproducibility of PSCs^19–22^. Recently, it was reported that the incorporation of ***rubidium*** cations (Rb^+^) into the A-site of the perovskite promoted the PCE to more than 21%, although the ionic radius of Rb^+^ (148 pm) was smaller than that of Cs^+^ (169 pm) and, according to the Goldschmidt’s tolerance factor, the formation of a stable perovskite structure was considered to be difficult^23,24^. The above findings motivated us to investigate ***potassium*** cations (K^+^) with an ionic radius (133 pm) for improving the photovoltaic performance of PSCs. Potassium is abundant in the earth’s crust (21,000 ppm), as compared to caesium (90 ppm) or rubidium (3 ppm), and it is available cheaply.

On the other hand, there is a big issue with *I-V* hysteresis of PSCs that causes uncertainty as to its real PCE^25–27^. Several hypotheses, such as ion migration^28,29^, polarisation^30^, defect traps^31^, and capacitance^32^, have been discussed to explain *I-V* hysteresis. In the conventional structure of PSCs, the difference of the conduction band edge between the TiO_2_ and the perovskite forms a small barrier at the interface, which retards the transportation of electrons from the perovskite to the TiO_2_^22,33,34^. As for the electron transport layer (ETL), when TiO_2_ with a relatively higher conduction band minimum (CBM) was replaced by SnO_2_ or Cl-caped TiO_2_ with relatively lower CBM^22,33,35^, the *I-V* hysteresis was diminished, indicating the disappearance of the electron transfer barrier^22,33,35^. These results suggested that the band engineering of the organometal halide perovskite by composition tuning is important for diminishing *I-V* hysteresis.

In this study, we explored the feasibility of incorporating K^+^ into the perovskite absorber. The results revealed that incorporating a small amount of K^+^ into the double organic cation perovskite absorber (FA_0.85_MA_0.15_Pb(I_0.85_Br_0.15_)_3_) improved the photovoltaic performance of PSCs significantly, and K^+^ incorporation diminished *I-V* hysteresis. To understand the mechanism of the phenomena, we investigated the influence under various K^+^ ratios in the perovskite absorber.

## Results and Discussion

### Crystal structure and optical properties of the K^+^–doped perovskite

**Figure 1:**
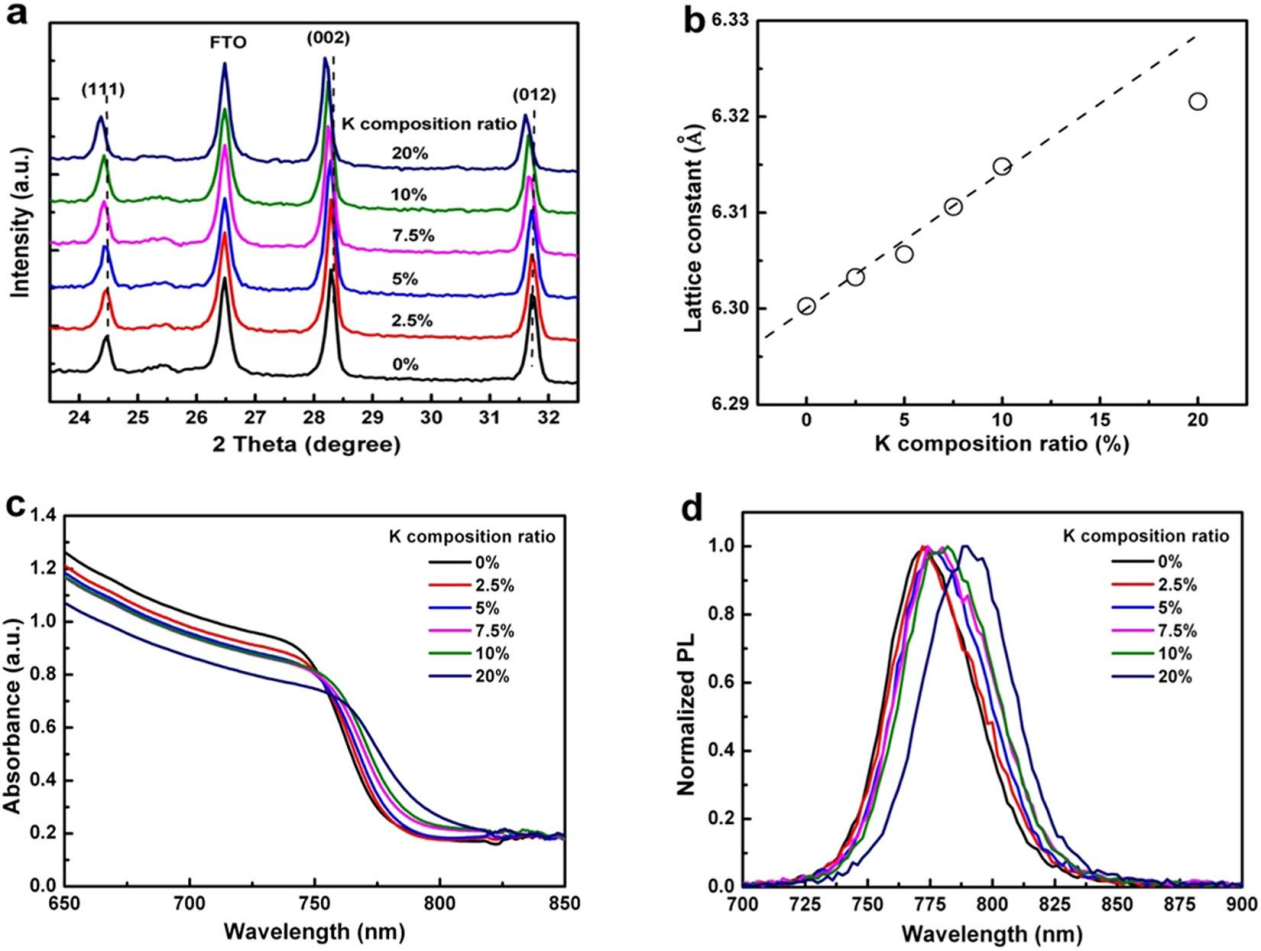
Properties of the different K^+^ ratio perovskite absorbers with a formula of Kx(FA_0.85_MA_0.15_)_1-x_Pb(I_0.85_Br_0.15_)_3_ (x = 0 to 0.2). (**a**) XRD patterns for perovskite of various K^+^ ratios. (**b**) Lattice constant as a function of the K^+^ ratio. (**c**) Absorption and (**d**) photoluminescence spectra for perovskite with various K^+^ ratios from 0% to 20%. The K^+^ ratio is defined by the molar ratio of K/(FA + MA + K). In the case of 0% K^+^, the perovskite is the double cation type, whose formula is FA_0.85_MA_0.15_Pb(I_0.85_Br_0.15_)_3_.

The crystal structure of the double organic cation perovskite of FA_0.85_MA_0.15_Pb(I_0.85_Br_0.15_)_3_ is trigonal^9,16^ or cubic^36,37^ at room temperature. To investigate the effect of incorporating K^+^ to the perovskite, XRD analysis was performed (Fig. 1a). The crystal lattice constant as a function of K^+^ composition (Fig. 1b) was calculated according to the 002 plane (2θ around 28.4°). The XRD peaks were shifted to a smaller angle when the K^+^ ratio was increased, indicating the elongation of crystal lattice, which was not observed for perovskite containing Cs^+^ and Rb^+^^20,24^. Considering Goldschmidt’s tolerance factor, the perovskite containing small cations would be collapsed. However, the K^+^–doped perovskite structure was kept as the cubic for a ratio of less than 10% K^+^ (Supplementary Figure 1a), suggesting that the K^+^ was incorporated into the crystal structure homogeneously. The δ-phase of FAPbI_3_ was not observed until 20% K^+^. On the other hand, the solubility limit of K^+^ in double-organic cation perovskite was over 20% K^+^ (Fig. 1b) where the non-photoactive phase of KPbI_3_^38^ might exist.

To investigate the effect of K^+^ incorporation on optical properties, the UV-vis absorption and PL spectra were recorded for perovskite prepared on FTO/mesoporous-TiO_2_ substrates. Figure 1c shows the absorption spectra for the perovskite with various K^+^ ratios. Obviously, the absorption edge shifted to a longer wavelength when the K^+^ ratio was increased, indicating the reduction of the band gap. Analogously, the normalised PL spectra for perovskite (Fig. 1d) showed that the peak shifted to a longer wavelength due to the decreasing band gap. Thus, the band gap of the perovskite absorber could be adjusted by the K^+^ ratio. The shrinkage of the band gap is good for longer wavelength absorption.

### Photovoltaic performance and *I-V* hysteresis

**Figure 2:**
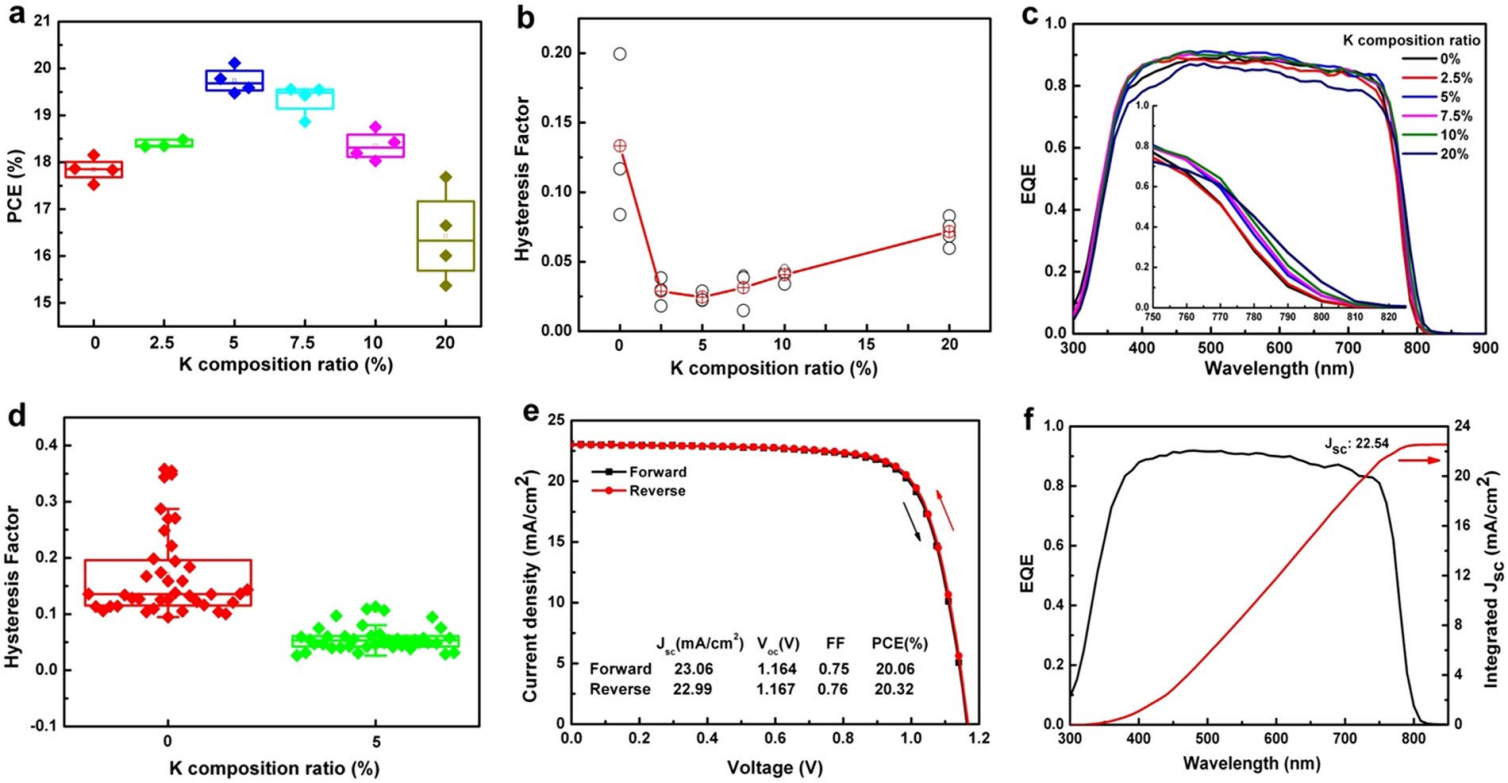
Photovoltaic performances of PSCs with various K^+^ ratios. (**a**) PCE for PSCs with various K^+^ ratios from 0% to 20%. (**b**) *I-V* hysteresis factor as a function of the K^+^ ratio. The dotted red circles denote the average value of 3 cells of the hysteresis factor on the respective K^+^ ratios. (**c**) The EQE spectra of PSCs with various K^+^ ratios from 0% to 20%. The inset shows enlarged spectra at the long wavelength tails. (**d**) Hysteresis factor statistics for 40 cells of PSCs without K^+^ and with 5% K^+^. (**e**) The *J-V* curve and (**f**) the EQE spectrum of the best PSC in this study.

PSCs with a mesoporous structure of FTO/compact TiO_2_:LiMg/mesoporous TiO_2_:Li/perovskite/Spiro-OMeTAD/Au (representative cross-sectional SEM image is shown in Supplementary Figure 2) were fabricated to investigate the influence of the K^+^ incorporation. Figure 2a shows the PCEs for the PSCs with several K^+^ compositions from 0 to 20%. Other photovoltaic parameters are shown in Supplementary Figure 3. It is clear that the maximum photovoltaic performance was realised at the 5% K^+^ ratio. The maximum short circuit current density (Jsc) and fill factor (FF) were obtained for the 5% K^+^ ratio, whereas the maximum open circuit voltage (Voc) was obtained for 2.5% K^+^ composition. Obviously, the *J*_sc_ presents a drop in the case of 2.5% K^+^. The incorporation of K^+^ results in the reduction of light absorption in the region of wavelengths shorter than 750 nm, which will lead to the decrease of *J*_sc_. Meanwhile, the absorption edge extends to longer wavelengths, which can cause the increase of *J*_sc_. These two factors compete and determine the overall *J*_sc_. In the case of 2.5% K^+^, the reduction of current density caused by the lower light absorption overwhelms the increase of current density originating from the extension of the absorption edge. Thus, the overall current density decreases. The *I-V* hysteresis factor, defined by (PCE_Reverse_ − PCE_Forward_)/PCE_Reverse_^39^, was estimated with various K^+^ ratios (Fig. 2b) where the most suppressed *I-V* hysteresis factor is obtained for 5% K^+^ ratio. It should be noted that *I-V* hysteresis was enhanced more than 10% at the K^+^ ratio, where it could be ascribed to phase separation. The external quantum efficiency (EQE) spectrums for solar cells with various K^+^ ratios from 0 to 20% are shown in Fig. 2c. Evidently, the decline at the longer wavelength, plotted in inset Fig. 2c, exhibits a red shift due to the shrinkage of the band gap with the increasing K^+^ ratio, which is consistent with the absorption and PL spectra.

To evaluate the reproducibility of PSCs without K^+^ and with 5% K^+^, forty cells were fabricated in each structure. The photovoltaic parameters are plotted in Supplementary Figure 4a–d, and the corresponding *I-V* hysteresis factors are plotted in Fig. 2d (J-V curves were recorded at 200 mV/s). It is obviously understood that *I-V* hysteresis is suppressed by the incorporation of K^+^, as the average hysteresis factor is 0.056, which is smaller than 0.171 without K^+^ incorporation. The averaged PCE for the 5% K^+^ ratio was 19.48%, including the highest efficiency of 20.32% and the lowest efficiency of 18.45%. Figure 2e and f shows the *J-V* curves at a scan rate of 200 mV/s and EQE spectrum of an efficient PSC, respectively. Both efficiencies calculated from forward and reverse scan were over 20%. The integrated current density (22.5 mA/cm^2^) obtained from the EQE spectrum is well matched to the value (22.9 mA/cm^2^) measured from the *J-V* curves.

To assess the effect of K^+^ incorporation on the *I-V* hysteresis of PSCs, the *J-V* curves without K^+^ and with 5% K^+^ were recorded at different scan rates (Supplementary Figure 5a and b); the corresponding *I-V* hysteresis factor versus scan rate is plotted in Supplementary Figure 5c. Obviously, *I-V* hysteresis is considerably larger in the device without K^+^. However, in the device with 5% K^+^, an *I-V* hysteresis factor of 0.006 is obtained, even at a high scan rate of 200 mV/s.

### Morphology of perovskite and photoluminescence (PL) decay

**Figure 3:**
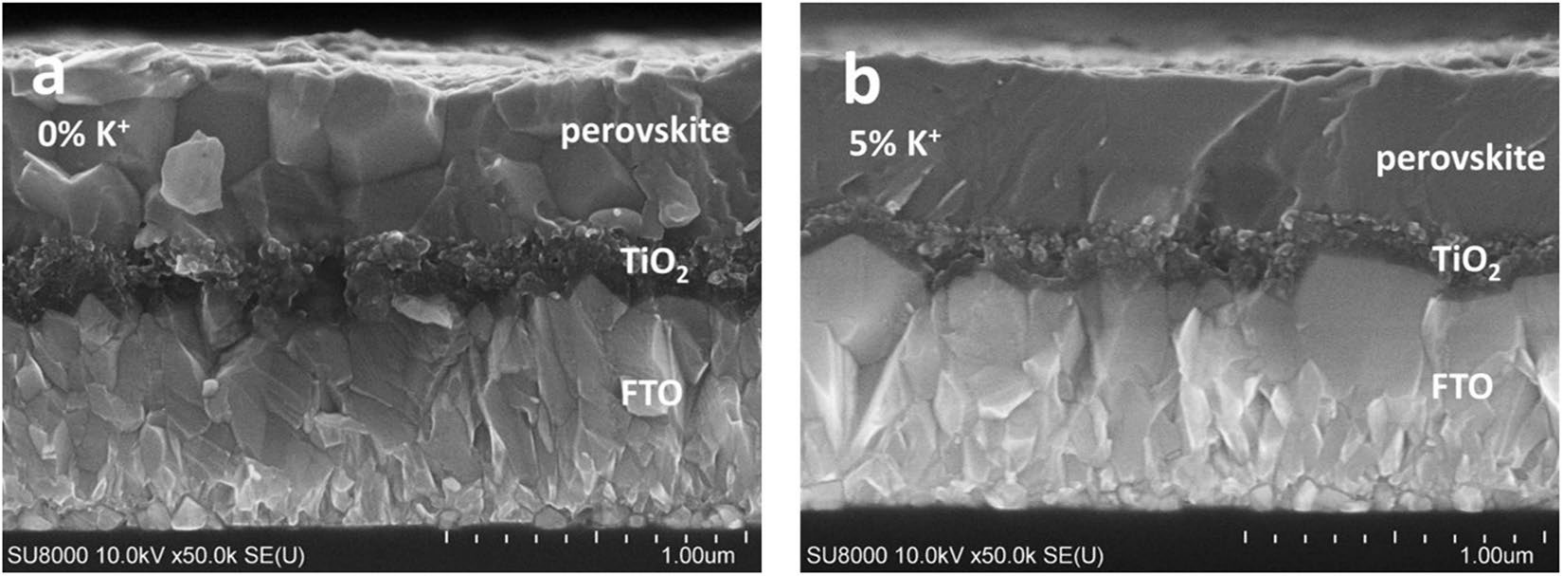
Cross-sectional SEM images for the PSCs. (**a**) PSC of perovskite without K^+^. (**b**) PSC of perovskite with 5% K^+^.

The effect of K^+^ on the morphology of the perovskite was investigated by SEM analysis. Surface SEM images for perovskite films with K^+^ ratios of 0 and 5% are shown in Supplementary Figure 6a and b, and the corresponding cross-sectional SEM images are shown in Fig. 3a and b. From the surface images, we find that the grains are tightly stacked and become larger with the incorporation of K^+^. Interestingly, in the cross-sectional SEM image, horizontal grain boundaries are apparent at the middle of perovskite layer without K^+^, whereas there are no horizontal grain boundaries for K^+^-doped perovskite absorber. The structure without horizontal grain boundaries would be favourable for carrier diffusion and superior photovoltaic performance.

To verify the influence of K^+^ incorporation in the perovskite absorber, the excited-state lifetimes of perovskites deposited on quartz or mesoporous TiO_2_ substrates were evaluated by time-resolved PL decay, as described in Supplementary Figure 7. The decay curve is analysed by a bi-exponential fitting^22^, and the corresponding parameters are listed in Supplementary Table 1. The estimated lifetime of perovskite on both substrates increased with incorporation of 5% K^+^, indicating a reduction of defects in the perovskite absorber. The excited state was quenched by the mesoporous TiO_2_ due to the injection of the electron. It is worth noting that electron transfer quenching was accelerated in the 5% K^+^-doped perovskite compared to quenching in the perovskite without K^+^.

### Investigation of the energy levels

**Figure 4:**
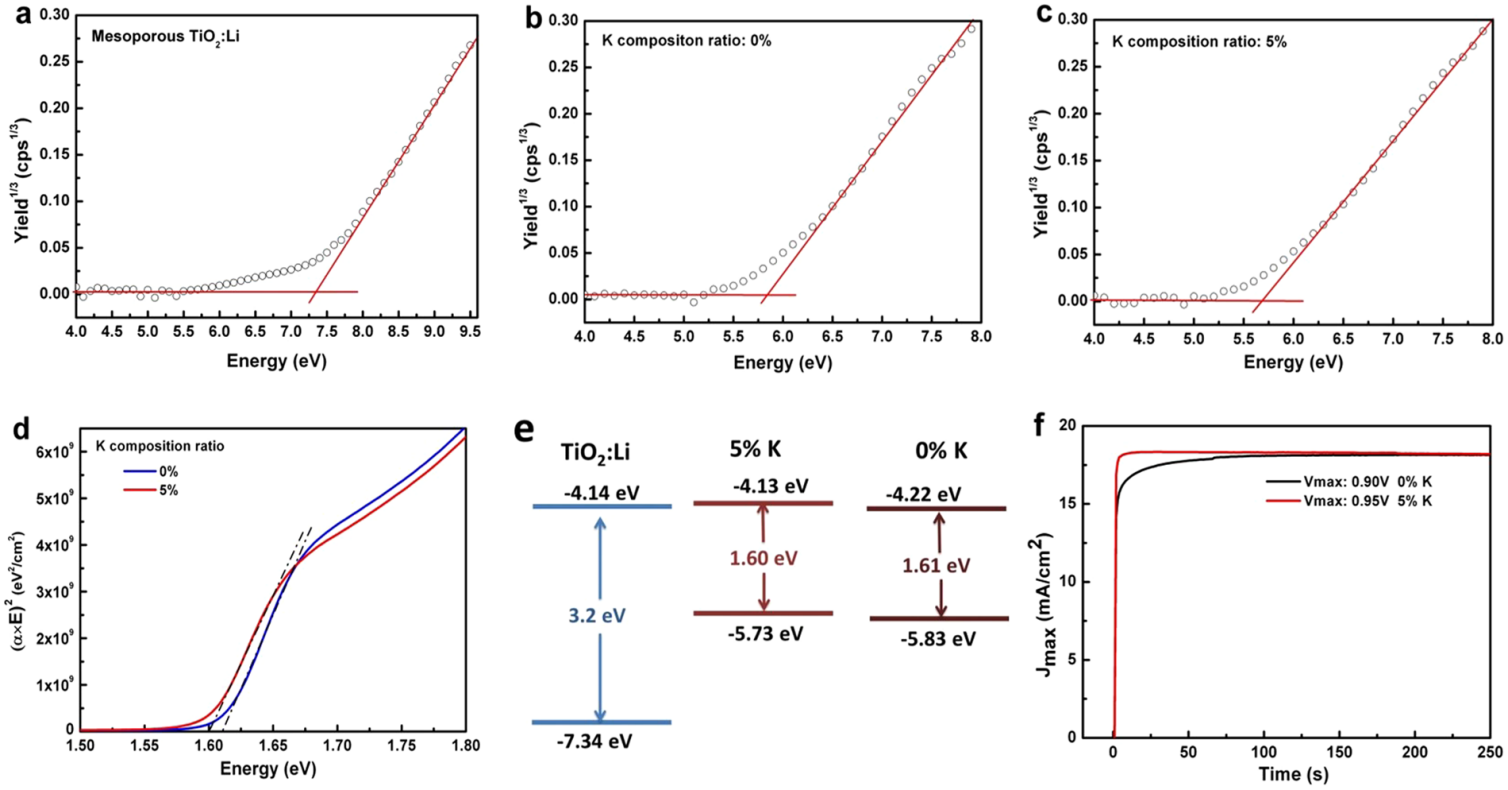
Ultraviolet photoelectron spectra, Tauc plots, energy levels, and photocurrent responses of related materials. Ultraviolet photoelectron spectra for (**a**) mesoporous TiO_2_:Li, (**b**) 0% K^+^ perovskite, and (**c**) 5% K^+^ perovskite. Estimated values are (**a**) −7.34 eV, (**b**) −5.83 eV, and (**c**) −5.73 eV. (**d**) Tauc plots of the perovskite with 5% K^+^ and without K^+^. Estimated band gaps are 1.61 eV for 0% K^+^ and 1.60 eV for 5% K^+^. (**e**) Energy levels of TiO_2_:Li, perovskite without K^+^, and perovskite with 5% K^+^. (**f**) Photocurrent response operated at maximal power point potential for PSCs using perovskite with 5% K^+^ and without K^+^.

To investigate the effect of K^+^ incorporation on energy levels, ultraviolet photoelectron spectra were measured for the TiO_2_:Li layer (Fig. 4a), the perovskite without K^+^ (Fig. 4b), and the perovskite with 5% K^+^ (Fig. 4c). Figure 4b and c revealed that the valence band maximum (VBM) of perovskite with a 5% K^+^ ratio shifted upward as compared to that without K^+^. The band gap energy with and without K^+^ was estimated from Tauc plots (Fig. 4d). The CBM of perovskite with a 5% K^+^ ratio, which was calculated from the VBM and the band gap energy, also shifted upward as compared to that of perovskite without K^+^. In summary, the band positions were estimated as shown in Fig. 4e. In the operating condition, excited electrons in perovskite diffuse to the ETL. When K^+^ is incorporated into perovskite, the CBM shifts upward, and the electron transfer barrier at the interface between the ETL and perovskite is reduced. The stagnation-less carrier transportation could minimise *I-V* hysteresis of PSCs. To verify the electron transport, the transient current rise of PSCs was measured for perovskite without K^+^ and perovskite with 5% K^+^. The transient current rise at the maximum power voltages of PSCs with 5% K^+^ was faster than that without K^+^, as shown in Fig. 4f. From different points of view, a simulation was performed investigating the electron transfer from perovskite to TiO_2_. An interfacial structure of perovskite/TiO_2_ was modelled; details of the method are provided in Supplementary Figure 8 and its note. The transition dipole moment indicates that the electron injection from the excited states of the perovskite to TiO_2_ was faster in the case of perovskite with K^+^ incorporated. Briefly, the disappearance of the barrier at the interface of TiO_2_ and the perovskite absorber is one reason the *I-V* hysteresis was suppressed in the PSCs with K^+^. It is worth noting that this finding points out a solution to suppress *I-V* hysteresis by modifying the perovskite absorber.

Finally, the stability of PSCs with K^+^ incorporated was evaluated. The solar cells were stored in a dark drying room (dewpoint temperature of −30 °C), and the *J-V* curves were recorded (Supplementary Figure 9). After 888 hours, the conversion efficiency maintained more than 90% of its initial performance, indicating a stable system in PCSs with K^+^ incorporated.

In summary, a small amount of K^+^ incorporated into the double organic cation perovskite absorber (FA_0.85_MA_0.15_Pb(I_0.85_Br_0.15_)_3_) was able to significantly improve the photovoltaic performance of PSCs and diminish *I-V* hysteresis. The incorporation of K^+^ into the perovskite absorber is an effective method for modifying the crystal lattice, decreasing its band gap, raising the position of the CBM more than TiO_2_, along with the vanishing of the grain boundary in it with a longer carrier lifetime. The disappearance of the energy barrier at the interface between TiO_2_ and perovskite with K^+^-incorporated absorbers is one reason for the elimination of *I-V* hysteresis. Eventually, more than 20% PCE without *I-V* hysteresis was achieved, and a future stable system is anticipated based on perovskite with K^+^ incorporated.

## Methods

### Materials

All chemicals were used as purchased without any purification. The materials for preparing the perovskite absorber, including PbBr_2_ (99%), MABr (>98%), and KI (>99.5%), were purchased from Tokyo Chemical Industry (TCI) Co., LTD. The PbI_2_ (99.99%) was obtained from Kojundo Chemical Laboratory Co., LTD. The FAI (>99%) was purchased from Dyesol, LTD. Titanium diisopropoxide bis(acetylacetonate) (75 wt. % in isopropanol), lithium bis(trifluoromethanesulfonyl)imide (LiTFSI), and 4-tert-butylpyridine (tBP) were purchased from Sigma-Aldrich. Magnesium(II) Bis(trifluoromethanesulfonyl)imide (Mg(TFSI)_2_) as a dopant in the compact layer was also obtained from TCI Co., LTD. TiO_2_ paste with a size of 24 nm was purchased from JGC C&C. The Spiro-OMeTAD was purchased from Merck. All solvents were purchased from Wako Pure Chemical Industries, LTD.

### Solar cell preparation

FTO glass with a sheet resistance of ~10 ohm/square from Nippon Sheet Glass Co. Ltd. was employed as a transparent conductive substrate. A series of cleanings using deionised water, detergent, acetone, methanol, and excimer was conducted sequentially. A 40-nm-thick compact layer with a composition of Ti_0.94_Li_0.03_Mg_0.03_O_2_ was then prepared by the spray pyrolysis method at 550 °C. LiTFSI and Mg(TFSI)_2_ were mixed in titanium diisopropoxide bis(acetylacetonate) as a precursor solution. A mesoporous TiO_2_ layer with a thickness of around 150 nm was then fabricated by spin coating with a TiO_2_ (PST-24NR)-based solution. Sintering treatment at 550 °C for 15 min produced the porous structure. Li-doping was conducted according to the literature; briefly, 0.1 M LiTFSI dissolved in acetonitrile was spin coated on the mesoporous layer after sintering at 450 °C for 30 min. A perovskite precursor solution containing 1.15 M PbI_2_, 0.20 M PbBr_2_, 0.20 M MABr, and 1.09 M FAI was prepared by dissolving all of the powders in DMF and DMSO mixed solvents with a volume ratio of 4:1. A KI precursor solution with a molar concentration of 1.5 M was prepared separately, and the desired volume was added into the above base precursor solution. Perovskite absorber layers were deposited based on the anti-solvent method, i.e. after dropping the precursor solution, a two-step spin coating with a rotation speed of 1000 rpm for 10 s and 4000 rpm for 30 s was conducted to disperse the droplets. One mL chlorobenzene was quickly dropped 20 s prior to the end of the program. The hole transport layer was then spin coated using Spiro-OMeTAD solution with a concentration of 72.25 mg/ml in chlorobenzene. To increase conductivity, LiTFSI (520 mg/mL in acetonitrile, 17.5 ul) and tBP (26.25 uL) were added. At last, a Au electrode (~100 nm) was deposited by thermal evaporation, and the area of the Au contact was more than 0.2 cm^2^. All processes were performed in a drying room with a dewpoint temperature of −30 °C.

### Characterisations

XRD characterisation was conducted on a Bruker D8 Discover diffractometer with Cu K-alpha radiation (λ = 0.15406 nm). Transmittance, reflectance, and absorbance were measured with a spectrophotometer (UV3600, Shimadzu). SEM images were measured with a field emission scanning electron microscope (SU8000, Hitachi). TR-PL was measured at room temperature using a 1-ns pulse width 532-nm Nd^+^ YAG laser and a time-correlated single photon counting system (C7990, Hamamatsu). Samples were excited with pulse energy of ~4.2 uJ/cm^2^. The valence band minimum was evaluated with a photoelectron spectrometer (BIP-KV201) developed by Bunkoukeiki Co., Ltd., Japan. The current density-voltage (*J-V*) curves were recorded under AM 1.5 G illumination (100 mW/cm^2^) with a 450-W xenon light source (YSS-80A; Yamashita Denso Co., Ltd., Japan). The light source was calibrated using a Si photodiode of BS-520 (Bunkoukeiki Co., Ltd., Japan). The scan speed was fixed at 200 mV/s, unless stated otherwise. The EQE spectrum was measured on an EQE system (CEP-2000MLQ, Bunkoukeiki Co., Ltd.) in the DC mode without any voltage bias. The excitation light intensity was calibrated using a Si photodiode. A black mask was used to confirm the photoactive area of 0.181 cm^2^ during *J-V* and EQE measurements.

### Simulation method

[(CH_3_NH_3_)_14_Pb_8_I_36_(Ti_84_O_181_H_30_)]^2−^ and [K(CH_3_NH_3_)_13_Pb_8_I_36_(Ti_84_O_181_H_30_)]^2−^ nanoclusters were used to model the perovskite/TiO_2_ interface. These nanoclusters were optimised using the semi-empirical PM6 method. The configuration interaction singles were used to calculate their excited states. All calculations were performed using a Gaussian09 software package.

## Electronic supplementary material

Supplementary information
