# Supplementary material for: Hysteresis-free perovskite solar cells made of potassium-doped organometal halide perovskite
Source: Sci Rep. 2017 Sep 22;7:12183. doi: 10.1038/s41598-017-12436-x (PMC5610231; doi:10.1038/s41598-017-12436-x)
Supplement: Supplementary file 1 — Supplementary information [file 41598_2017_12436_MOESM1_ESM.docx]

**Supplementary information**

**Hysteresis-free perovskite solar cells made of potassium-doped organometal halide perovskite**

Zeguo Tang,^1*^ Takeru Bessho,^1*^ Fumiyasu Awai,^2^ Takumi Kinoshita,^1^ Masato M. Maitani,^1^ Ryota Jono,^1^ Takurou N. Murakami,^3^ Haibin Wang,^1^ Takaya Kubo,^1^Satoshi Uchida,^1^ & Hiroshi Segawa^1,^ ^2*^

^1^ Research Center for Advanced Science and Technology (RCAST), The University of Tokyo, 4-6-1, Komaba, Meguro-ku, Tokyo 153-8904, Japan

^2^ Graduate School of Arts and Sciences, The University of Tokyo, 3-8-1, Komaba, Meguro-ku, Tokyo 153-8902, Japan

^3^ Research Center for Photovoltaics, National Institute of Advanced Industrial Science and Technology (AIST), 1-1-1 Higashi, Tsukuba, Ibaraki 305-8565, Japan

Corresponding author: Hiroshi Segawa, [csegawa@mail.ecc.u-tokyo.ac.jp](mailto:csegawa@mail.ecc.u-tokyo.ac.jp)

**
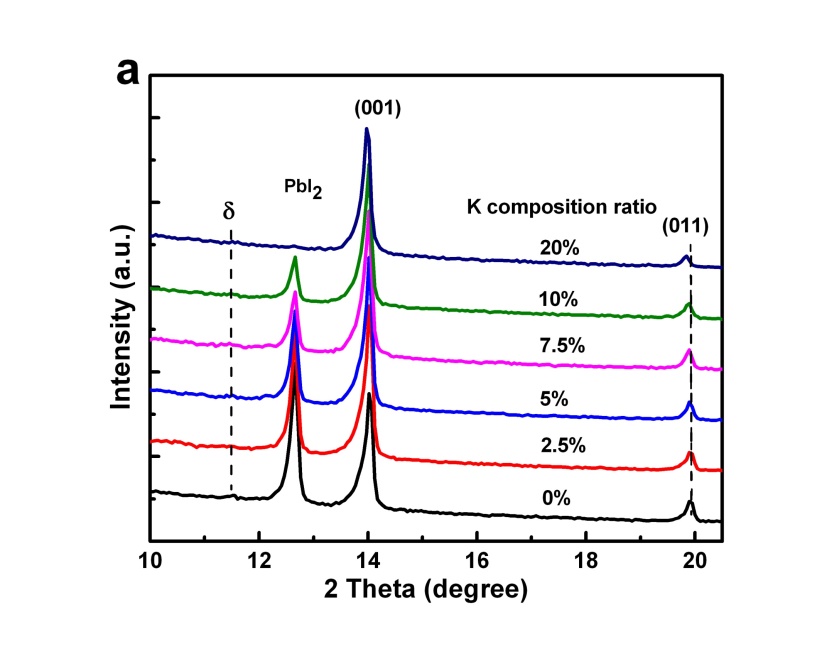

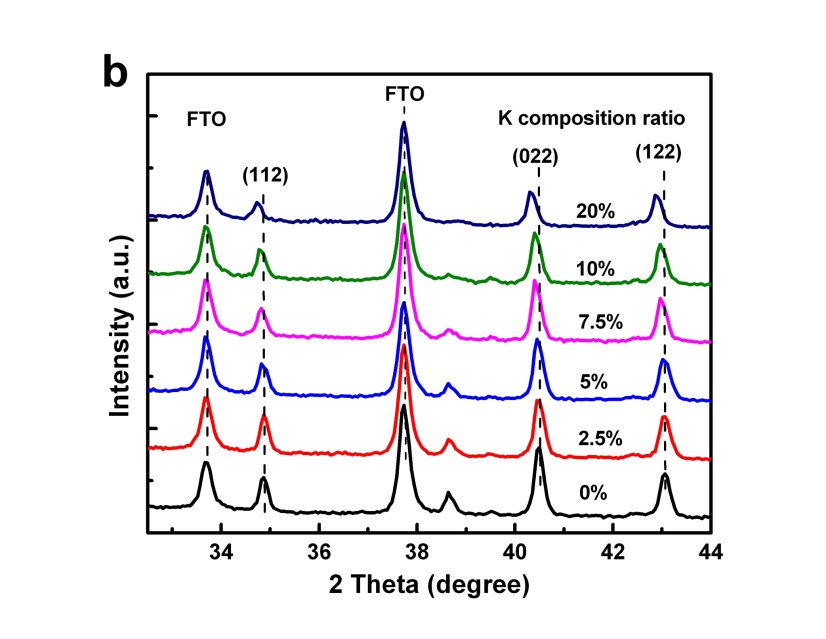
**

**Supplementary Figure 1. XRD patterns for perovskite absorbers with different K composition ratios.** XRD patterns in the two theta region of (a) 10º to 20.5º and (b) 32.5º to 44º for perovskite absorbers with K composition from 0% to 20%. The K composition ratio is defined by the mole ratio of K/(FA+MA+K). A 0% K case means the double cation perovskite of FA_0.85_MA_0.15_Pb(I_0.85_Br_0.15_)_3_ K mixed perovskites with a formula of K_x_(FA_0.85_MA_0.15_)_1-x_Pb(I_0.85_Br_0.15_)_3_ (x = 0 to 0.2 in this study).


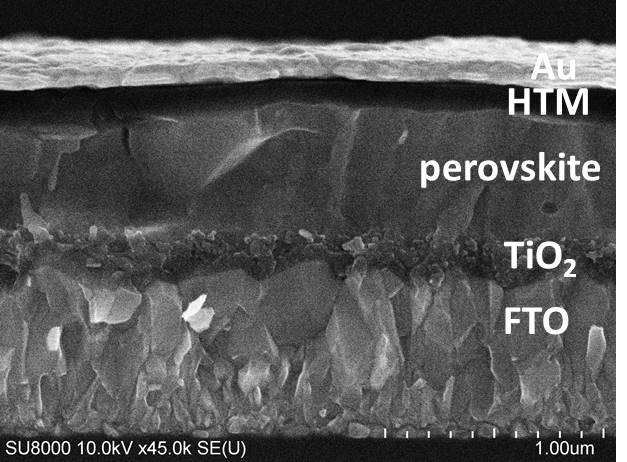


**Supplementary Figure 2. Representative cross-sectional SEM image of PSCs.** The device structure layered with FTO/TiO_2_ under layer/TiO_2_ mesoporous layer with Li doped/perovskite absorbers/hole transport layer/gold.

**
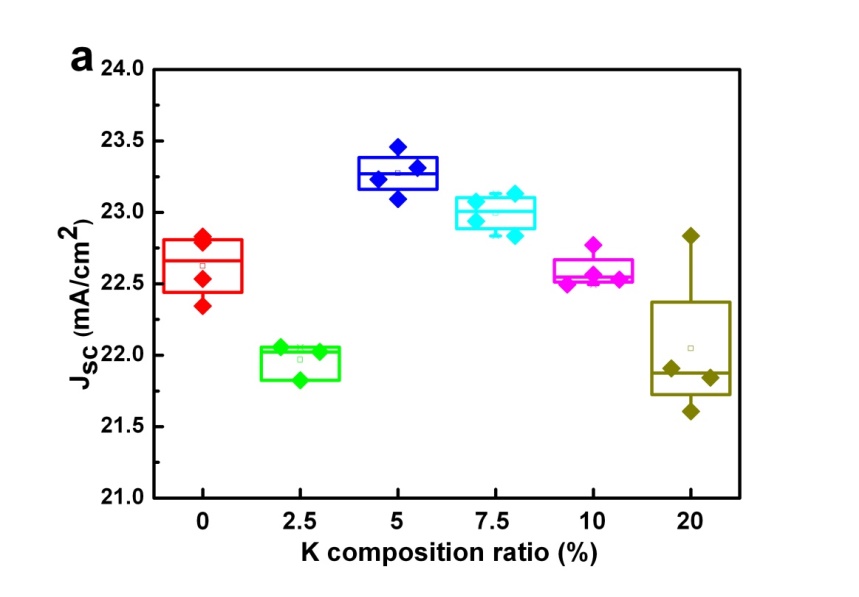
**

**
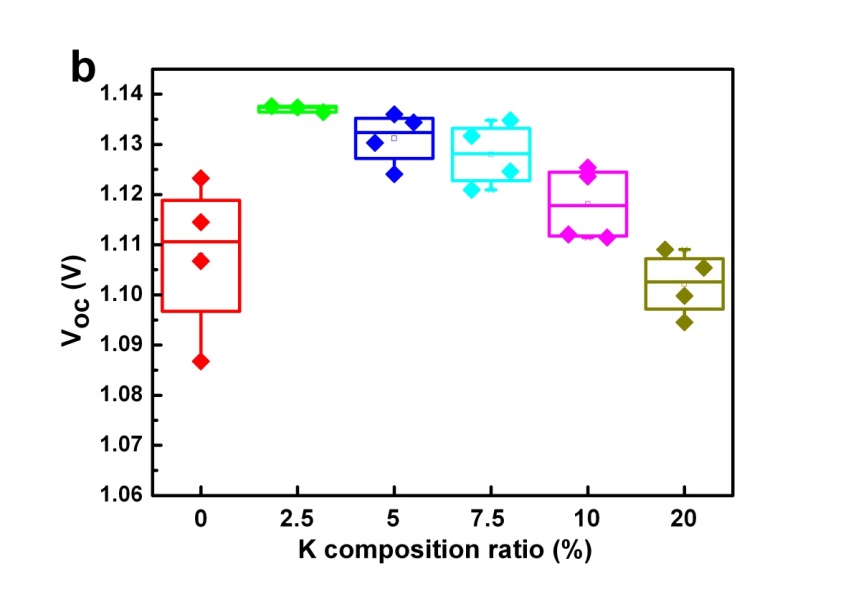
**

**
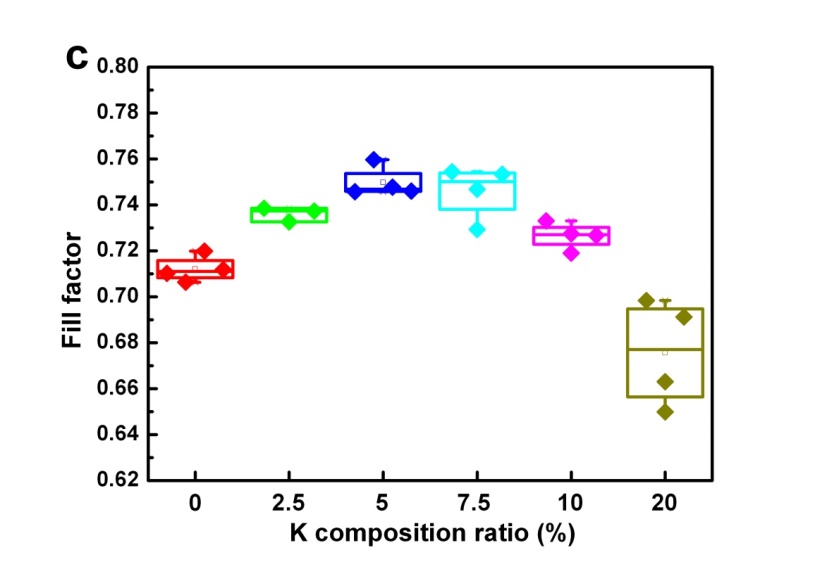
**

**Supplementary Figure 3. Photovoltaic performances of PSCs with K ratios from 0% to 20%.** (a) Short-circuit current density, (b) open-circuit photo voltage, and (c) fill factors are described.


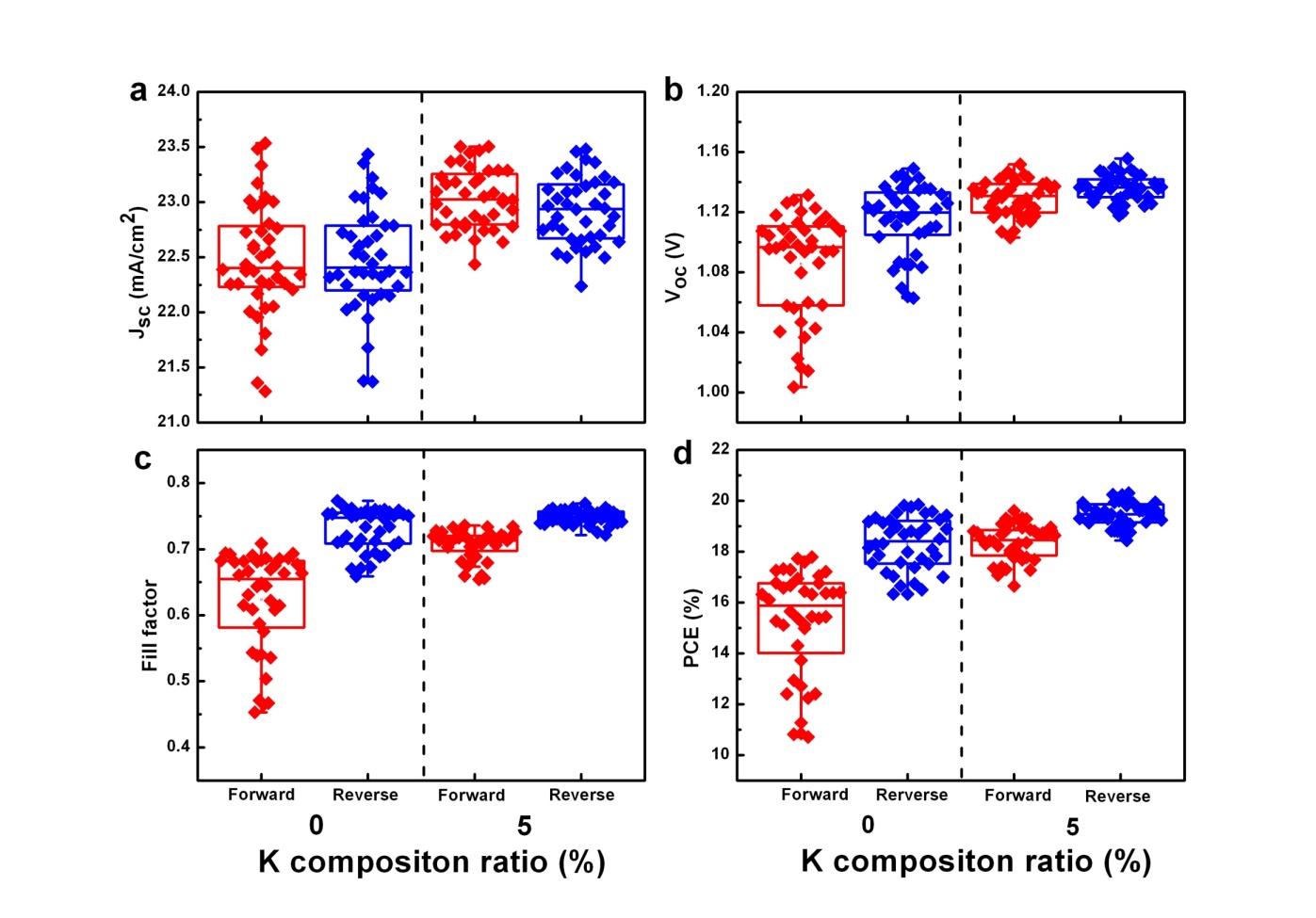


**Supplementary Figure 4. Photovoltaic performances for 40 cells without K and with a 5% K ratio.** In comparison, *J*_sc_, *V*_oc_, fill factor, and PCE were described as (a), (b), (c), and (d), respectively.

**
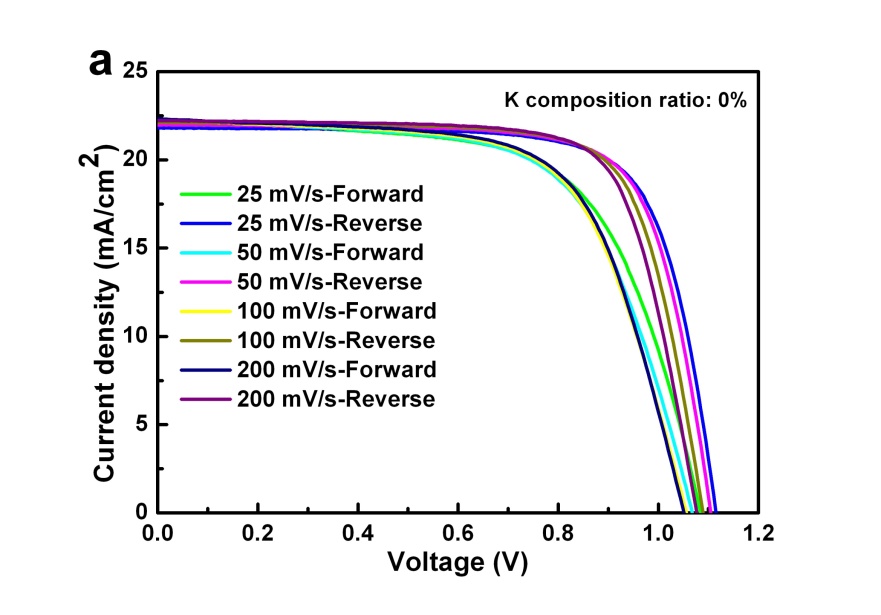
**

**
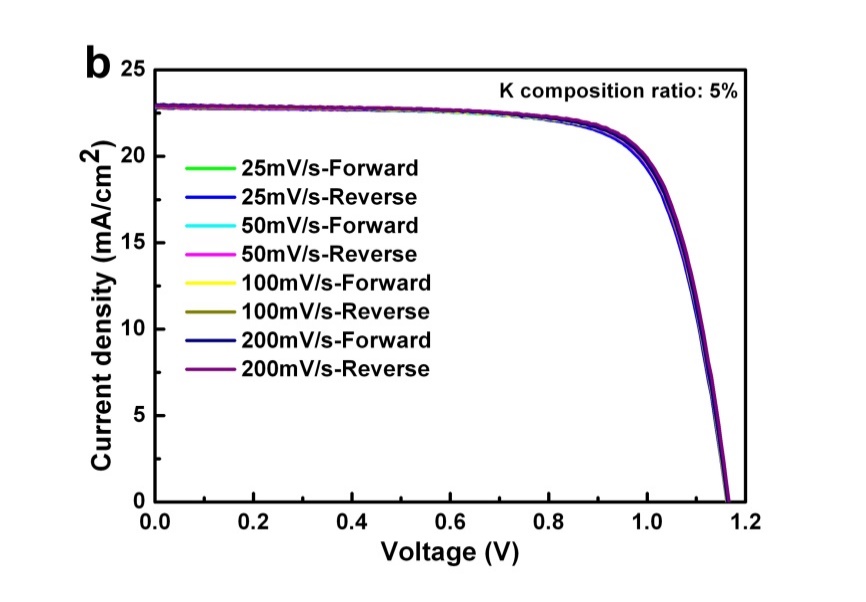
**

**
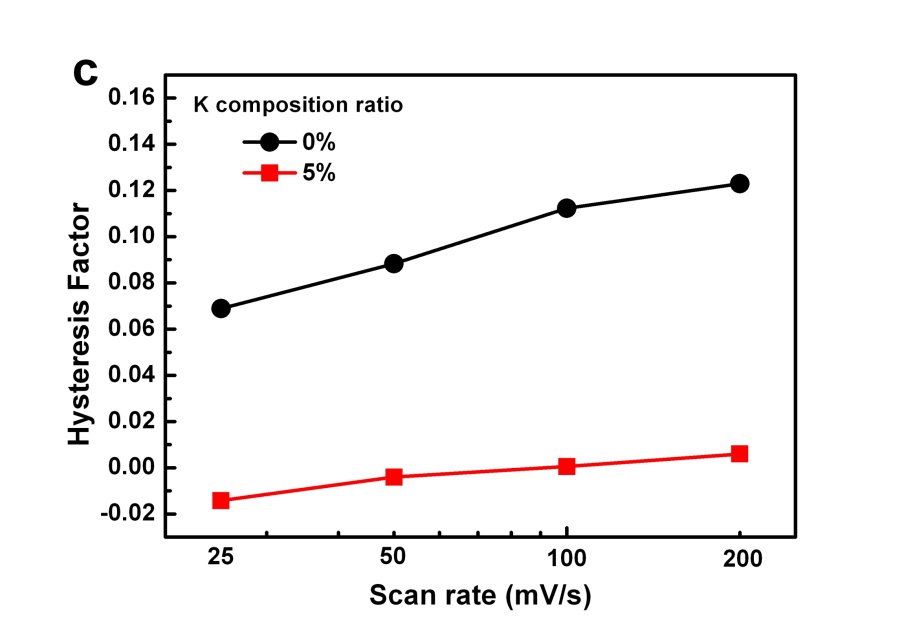
**

**Supplementary Figure 5. *J-V* curves and hysteresis factors.** Recorded at different scan rates for PSCs (a) without K and (b) with a 5% K ratio. (c) Relationship between hysteresis factors as a function of the scan rates for PSCs without K and with a 5% K ratio.

**
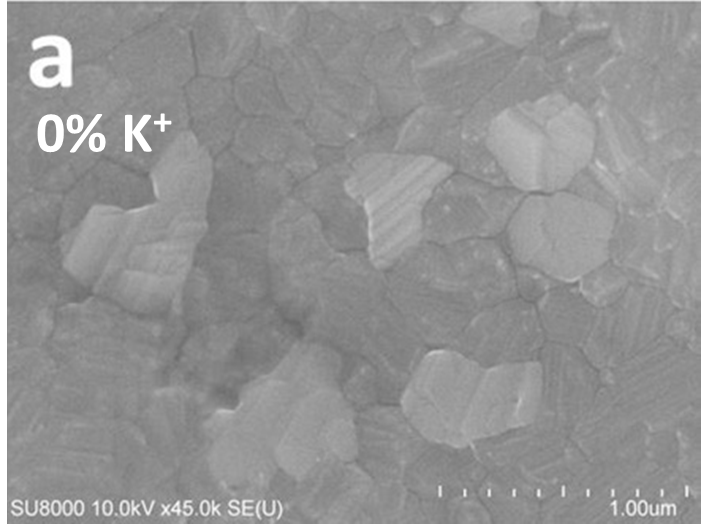
**

**
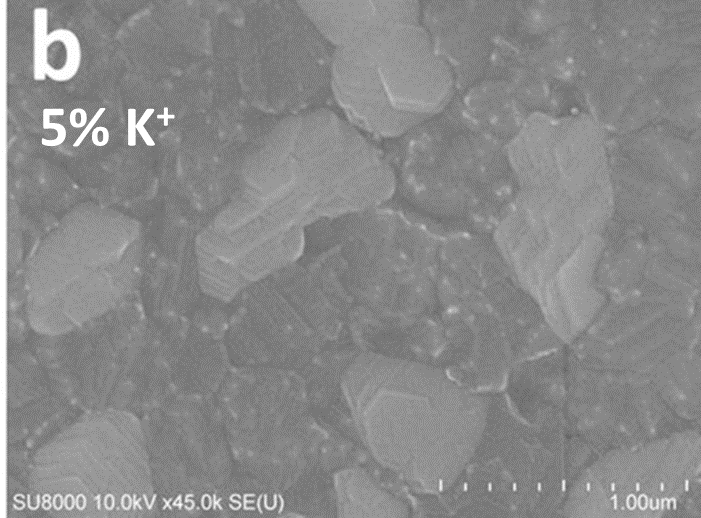
**

**Supplementary Figure 6. Surface analysis via scanning electron microscopy (SEM).** The images for perovskite absorbers (a) without K and (b) with a 5% K ratio on a 1-μm scale.


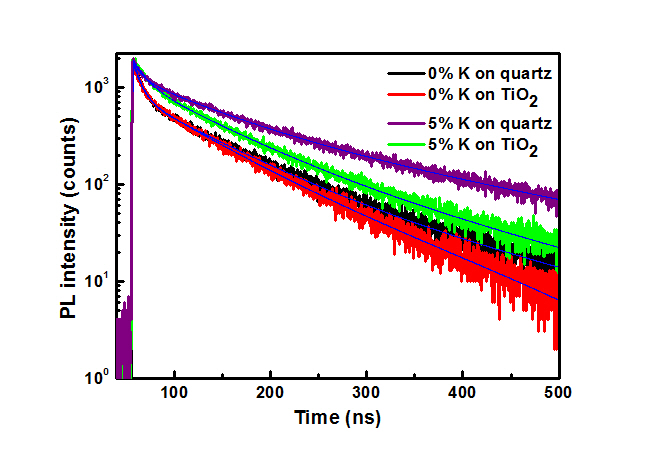


**Supplementary Figure 7. PL decay curves.** The sample was fabricated as perovskite absorbers on quartz and mesoporous TiO_2_ substrates without K and with a 5% K ratio.

$$A= A_{1}*e^{-t/\tau_{1}}+ A_{2}*e^{-t/\tau_{2}}$$

| Samples | τ_1_ | τ_2_ | A_1_ | A_2_ | A_1_/(A_1_+A_2_) | A_2_/(A_1_+A_2_) |
| --- | --- | --- | --- | --- | --- | --- |
| 0% K on quartz | 11.2 | 107.9 | 1191.6 | 634.4 | 0.65 | 0.35 |
| 0% K on TiO_2_ | 11.0 | 92.4 | 1001.3 | 697.7 | 0.59 | 0.41 |
| 5% K on quartz | 32.7 | 173.5 | 789.5 | 821.8 | 0.49 | 0.51 |
| 5% K on TiO_2_ | 23.9 | 118.2 | 991.4 | 802.1 | 0.55 | 0.45 |

**Supplementary Table 1. The fitting parameters of PL decay.** Fitting was conducted following a bi-exponential equation for perovskite absorbers with K ratios of 0% and 5% on quartz and mesoporous TiO_2_ substrates, respectively.

**Supplementary Note.** To confirm the energy position at the perovskite/TiO_2_ interface, we modelled interfacial structures using a pseudo-cubic Pb8 nanocluster on the (101) facet of the anatase Ti84 nanocluster. The [(CH_3_NH_3_)_14_Pb_8_I_36_(Ti_84_O_181_H_30_)]^2−^ and [K(CH_3_NH_3_)_13_Pb_8_I_36_(Ti_84_O_181_H_30_)]^2−^ nanoclusters were used to model the perovskite/TiO_2_ interface. The MA^+^ and K^+^ ions were placed in the position of the A-site cation in a pseudo-cubic framework. The highest occupied molecular orbital (HOMO) and the lowest unoccupied molecular orbital (LUMO) of the total system were localised around the pseudo-cubic perovskite moiety and the anatase TiO_2_ nanocluster moiety, respectively. Configuration interaction calculations showed strong allowed transitions in 2.18 eV for the MA^+^ cation system and 2.16 eV for the K^+^ cation system. These transitions were attributed to local excitations in the pseudo-cubic perovskite moieties. On the other hand, the oscillator strengths of the charge-separated states were very weak because electronic coupling between the system’s HOMO and LUMO was very small. The energy differences between the charge-separated states, which were the conduction band minimum of the TiO_2_ moiety with an electron transferred from perovskite absorbers and their ground states for the MA^+^ and K^+^ cation systems, corresponded to 2.18 eV and 2.14 eV, respectively. The transition dipole moment between excited states indicated that the electron injection from the excited states of the perovskite moiety to the conduction band of the TiO_2_ moiety should be very fast because their electronic coupling was very strong. The energy differences from the TiO_2_ conduction band edge to the MA^+^ and K^+^ cation systems were 0.00 eV and 0.02 eV, respectively.


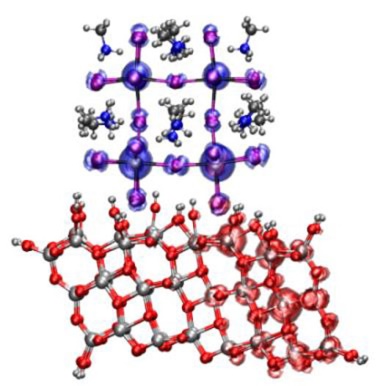


**Supplementary** **Figure 8. [(CH_3_NH_3_)_14_Pb_8_I_36_(Ti_84_O_181_H_30_)]^2−^ model for simulating the TiO_2_/perovskite** **interface**

|  | MAPbI_3_ | K_x_(MAPbI_3_)_1-x_ |
| --- | --- | --- |
| *E*_CS_/eV | 2.18 | 2.14 |
| *E*_LE_/eV | 2.18 | 2.16 |
| *E*_LE_-*E*_CS_/eV | 0 | +0.02 |
| $\left\langle\Psi_{\mathrm{LE}} \vert\hat{V} \vert\Psi_{\mathrm{CS}} \right\rangle$/eV | 0.1 | 0.2 |

**Supplementary Table 2. Simulation result for the TiO_2_/perovskite interface.**

*E*_CS_ and *E*_LE_ represent the transition from perovskite to TiO_2_ and the transition from perovskite to perovskite, respectively. $\left\langle\Psi_{\mathrm{CS}} | \hat{V} | \Psi_{\mathrm{LE}} \right\rangle$ is the electronic coupling of CS and LE.

**
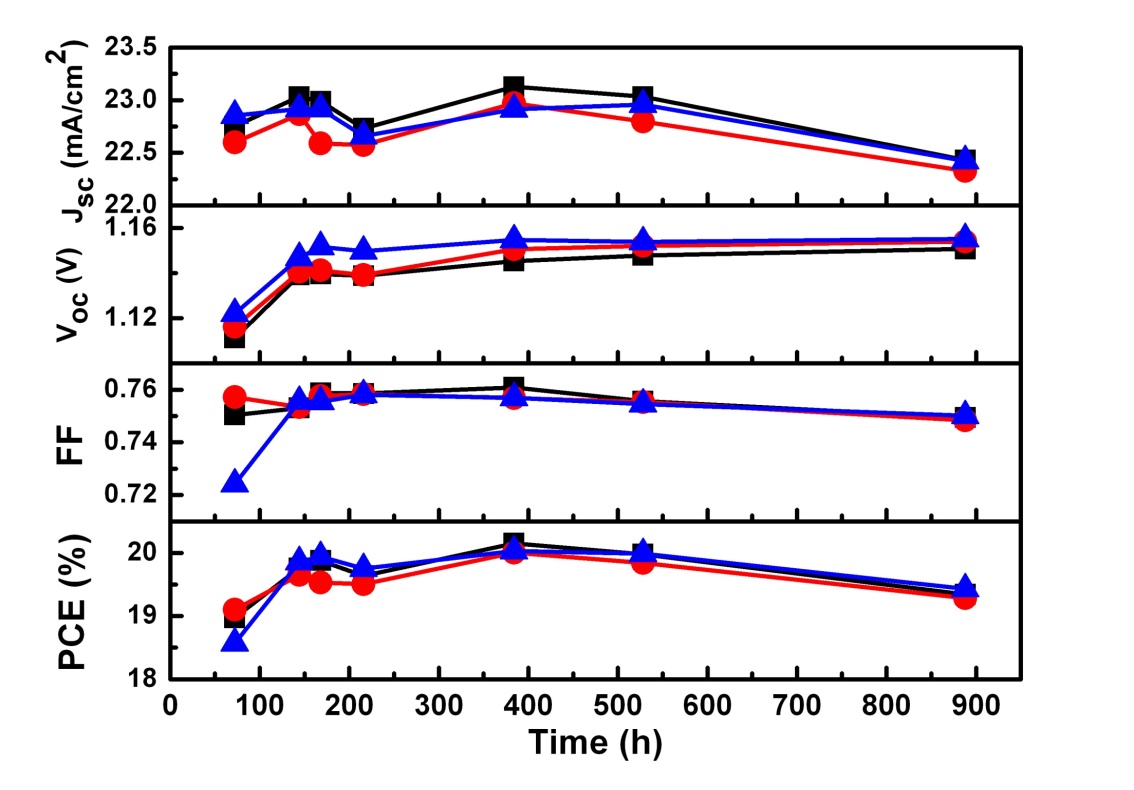
**

**Supplementary Figure 9. Stability test for three PSCs with 5% K composition ratios**
